# Prevalence of Clinical and Pre-Clinical Obesity at Six Months Postpartum Following Gestational Diabetes Mellitus

**DOI:** 10.3390/nu18020212

**Published:** 2026-01-09

**Authors:** Cristina Gómez Fernández, Laura A. Magee, Marietta Charakida, Tanvi Mansukhani, Peter von Dadelszen, Cristina Fernández Pérez, Francesco Rubino, Kypros H. Nicolaides

**Affiliations:** 1 Harris Birthright Research Centre for Fetal Medicine, King’s College Hospital, London SE5 8BB, UK; cgfernandez@torrejonsalud.com (C.G.F.); marietta.charakida@kcl.ac.uk (M.C.); tanvi.mansukhani@nhs.net (T.M.); 2 Faculty of Medicine, Complutense University of Madrid, 28040 Madrid, Spain; 3 Department of Women and Children’s Health, School of Life Course and Population Sciences, King’s College London, London SE1 1UL, UK; laura.a.magee@kcl.ac.uk (L.A.M.); pvd@kcl.ac.uk (P.v.D.); 4 School of Biomedical Engineering and Imaging Sciences, King’s College London, London WC2R 2LS, UK; 5 2nd Department of University Paediatric Clinic, National and Kapodistrian University of Athens, 10679 Athens, Greece; 6 Preventive Medicine Department (IDIS), Santiago de Compostela University, 15706 A Coruña, Spain; cristina.fernandez.perez3@sergas.es; 7 Faculty of Life Sciences and Medicine, School of Cardiovascular and Metabolic Medicine and Sciences, King’s College London, London SE1 1UL, UK; francesco.rubino@kcl.ac.uk

**Keywords:** postpartum, gestational diabetes, obesity, metabolic disease

## Abstract

**Background/Objectives**: A number of initiatives have refocused attention from obesity to adiposity-related organ dysfunction. In this prospective observational study, we examined this paradigm postpartum. **Methods**: At King’s College Hospital, London, UK, we invited for review by six months postpartum, consecutive women with GDM (N = 1442, September 2023–August 2025) and without GDM (N = 646, January 2025–August 2025). Those with excess adiposity (BMI ≥ 30 kg/m^2^ and waist-to-height ratio > 0.5) were assessed for organ dysfunction, using criteria from a recent Commission: anovulation, metabolism or renal clusters, raised blood pressure, or elevated end-diastolic left ventricular filling pressure. Multiple regression determined predictors of adiposity-related organ dysfunction, the prevalence of which was calculated as a range (highest estimate: absolute organ dysfunction prevalence; lowest estimate: adiposity-adjusted, as highest estimate minus prevalence of organ dysfunction in women without excess adiposity). **Results**: Of those invited for review, 1086/1442 (75.3%) GDM and 562/646 (87.0%) non-GDM women attended, at median 5.8 months after birth (interquartile range 4.8–6.7). Excess adiposity was observed in 385/1086 (35.5%) GDM and 117/562 (20.8%) non-GDM women, among whom organ dysfunction was seen in 61.0% GDM (235/385), 51.3% non-GDM (60/117). 35.9% (408/1137) of women without excess adiposity. Organ dysfunction attributable to excess adiposity was estimated to be 22.9% (58.8% minus 35.9%), and was poorly predicted by the multivariable model (AUC 0.64, 95%CI 0.60–0.69). **Conclusions**: Among women with prior GDM, organ dysfunction attributable to excess adiposity affects at least 20% of those with excess adiposity postpartum, and is not currently predictable.

## 1. Introduction

A number of initiatives have refocused attention from obesity, to the adiposity-related impact on health. These initiatives include the Edmonton Obesity Staging System (EOSS) for clinical severity [1], the European Association for the Study of Obesity (EASO) [2], and a recent Lancet Diabetes and Endocrinology Commission for clinical obesity [3]. All consider the medical and metabolic impact of excess adiposity on health, but the Commission proposed specific criteria, representing obesity-related limitations of daily activities or organ dysfunction, including functional alterations within cardio-pulmonary, reproductive, metabolic, nervous, lymphatic and musculoskeletal systems, due to mechanical, inflammatory, or endocrine mechanisms of obesity pathophysiology.

As with other chronic illnesses, obesity management aims to achieve timely access to evidence-based medical or surgical treatment of obesity that is affecting health, to improve or resolve those clinical manifestations. New initiatives (as above) aim to personalize and risk-stratify care, whilst recognizing that obesity is still associated with an increased risk of adiposity-related chronic diseases (e.g., cancer), that risk varies between individuals according to other factors, and appropriate care may vary from standard lifestyle counselling and monitoring over time (if the risk is low), to active treatment (if the risk is higher, or to facilitate treatment of other diseases that may be undermined by the presence of obesity, such as type 2 diabetes).

The metabolic changes associated with obesity (i.e., insulin resistance affecting glucose, lipid, and protein metabolism [4,5]) increase the risk of gestational diabetes mellitus (GDM, impaired glucose tolerance that is first diagnosed during pregnancy [6]), and other maternal and newborn complications for mothers and babies [6,7] (such as hypertension, stillbirth, and large-for-gestational age infants and associated birth trauma). As these women have a ten-fold higher risk of future pre-diabetes or type 2 diabetes [8], and more than a two-fold higher risk of cardiovascular events in the first decade postpartum [9], we have prospectively followed a cohort of women with GDM (and non-GDM controls), and assessed many criteria for adiposity-related organ dysfunction.

In this study, we estimate the prevalence of obesity-related organ dysfunction postpartum among women with GDM in their most recent pregnancy (vs. non-GDM controls), as they are at potentially higher risk of cardio-renal-metabolic forms of adiposity-related organ dysfunction.

## 2. Materials and Methods

### 2.1. Characteristics of the Cohort

At the Fetal Medicine Unit of King’s College Hospital, London, UK, all pregnant women attend routine fetal ultrasound examinations at 12-, 20-, and 36 weeks’ gestation. At their 36-week ultrasound visit, since September 2023, we have invited all women with GDM to attend a postnatal review clinic by six months after birth, to assess their cardiovascular and metabolic health. Strategies to maximize attendance have included: recruiting at the routine 36 week’s visit for ultrasound examination; providing women with a wide range of appointment options; inviting them to attend the appointment with partners, friends, and/or children; and contacting women 1–2 days before the planned visit, to give them the option of rescheduling. Also, since January 2025, we have extended this invitation for postnatal review at six months postpartum, to women without GDM who were attending the same antenatal care setting, and could act as controls for our GDM cases.

In the UK, screening and diagnosis of GDM is according to National Institute for Health and Care Excellence (NICE) guidance, which advises that pregnant women undergo GDM risk assessment in the first trimester, using the following clinical risk factors: ethnicity associated with a high prevalence of GDM (e.g., South Asian or Black ethnicity), BMI ≥ 30 kg/m^2^, macrosomia or GDM in prior pregnancy(ies), and family history (first-degree relative) of diabetes mellitus [10]. Women with one or more of these risk factors are offered biochemical testing, usually by the 75 g 2 h oral glucose tolerance test (OGTT) at 24–28 weeks’ gestation, but with prior GDM specifically, either early pregnancy OGTT or blood glucose self-monitoring is offered. GDM is diagnosed if fasting plasma glucose is ≥5.6 mmol/L or 2 h plasma glucose level is ≥7.8 mmol/L [10], or if blood glucose self-monitoring is abnormal (e.g., fasting ≥5.3 mmol/L, or 1 h post-meal ≥ 7.8 mmol/L). Once GDM is diagnosed, women are offered education and intervention to reduce blood glucose, consisting of diet and activity, with/without additional metformin or insulin, as needed.

### 2.2. Ethics

Women gave written informed consent to participate in the study, which was approved by the National Health Service (NHS) Research Ethics Committee (reference 18/NI/0013; Integrated Research Application System ID 237936).

### 2.3. Clinical Assessments at Postnatal Study Visit

At the postnatal visit, the following information was recorded: (i) maternal demographics and medical history, including maternal age and self-reported ethnicity, method of conception, family history of diabetes mellitus (first- or second-degree relative), chronic hypertension and whether they had more pregnancies than the recent one or not; and (ii) details of the most recent pregnancy, including GDM, gestational hypertension or pre-eclampsia, gestational age at birth, and birthweight; and (iii) current medications, including contraceptives, and drugs to treat hyperglycaemia, hypertriglyceridemia, hypercholesterolaemia, or elevated blood pressure (BP). Assessments of body weight and adiposity included weight (kg), height (m), systolic and diastolic BP (mmHg), and waist circumference (cm). BMI was calculated (as weight in kg, divided by height^2^ in m^2^) and considered abnormal if ≥30.0 kg/m^2^. The waist-to-height ratio (WHR) was calculated and considered abnormal if >0.5 [3].

The following tests were performed: 75 g 2 h OGTT [11] (interpretation, as above [10]), HbA1c (mmol/mol), serum triglycerides (mmol/L), serum high-density lipoprotein cholesterol (mmol/L), serum creatinine (µmol/L), urine albumin/creatinine ratio (uACR, mmol/mol), and maternal echocardiography (as previously described [12]). All laboratory assays were performed according to standard operating procedures.

Data on baseline maternal and pregnancy characteristics and outcomes (including gestational hypertension and pre-eclampsia [13], gestational age at birth, and birthweight) were collected (or verified) from hospital maternity records or from women’s general practitioners.

### 2.4. Assessment of Obesity Status, Clinical and Pre-Clinical Obesity

We used two measures of body size for confirmation of excess adiposity: postnatal BMI and WHR. WHR accounts for general body size, in contrast to waist circumference alone; WHR is strongly related to visceral adiposity, and it is the simplest predictor of hypertension, diabetes, and cardiovascular disease, which were of particular interest in our study population [14]. More direct measures of body fat were not available (e.g., subcutaneous fat by skinfold callipers, bioelectrical impedance analysis, or the gold standard of dual-energy X-ray absorptiometry [15]). Consistent with the Commission’s recommendations, we defined (confirmed) obesity as BMI ≥ 30.0 kg/m^2^ and WHR > 0.5. Since we did not have access to direct body fat measurement or other measures of body size beyond WHR, we defined the absence of obesity as BMI < 30 kg/m^2^.

The outcome of primary interest was ‘clinical obesity’, defined as excess adiposity plus one/more organ dysfunction criteria or limitations of daily activity, as defined by the Commission [3]. Pre-clinical obesity was defined as excess adiposity without these criteria.

Information was obtained about the 17 criteria that allow diagnosis of clinical obesity in adult women [3], with the exception of sleep-disordered breathing due to increased upper airways resistance, chronic urinary incontinence, and non-alcoholic fatty liver disease with fibrosis. The remaining 14 criteria defined by the Commission were: intracranial hypertension; reduced lung and diaphragmatic compliance causing respiratory problems; heart failure, systolic or diastolic (increased left ventricular filling pressure [E/e’] > 90th percentile, as 9.6); atrial fibrillation; pulmonary hypertension; recurrent venous thromboembolism; ‘raised arterial blood pressure’ (BP); ‘metabolism’ cluster (hyperglycaemia, high triglycerides, and low HDL); ‘renal’ cluster (microalbuminuria and reduced estimate glomerular filtration rate [eGFR]); anovulation, oligo-menorrhea and polycystic ovarian syndrome (as assisted reproductive technology); musculoskeletal problems; lymphoedema; or limitations of activities of daily living. Definitions of the components of the metabolism cluster are listed in Box 1. For the renal cluster, microalbuminuria was uACR ≥ 3 mg/mmol, and eGFR < 90 mL/min/1.73 m^2^, calculated by the 2009 Chronic Kidney Disease-Epidemiology Collaboration without the race coefficient, to align with current NICE guidance [16,17]. Raised BP cluster was Stage 1 hypertension (systolic 130–139 mmHg or diastolic BP 80–89 mmHg), Stage 2 hypertension (systolic ≥ 140 mmHg or diastolic BP ≥ 90 mmHg) [18], or taking antihypertensive treatment, regardless of BP.

Box 1. **Definition of ‘metabolism’ cluster for clinical obesity in adults.**

Hyperglycaemia was defined as meeting criteria for prediabetes or DM, according to the American Diabetes Association. Prediabetes was defined as one/more of: HbA1c 39–47 mmol/mol, fasting plasma glucose 5.6–6.9 mmol/L, or impaired glucose tolerance as 2 h blood glucose from an oral glucose tolerance test (OGTT) of 7.8–11 mmol/L. Type 2 diabetes was defined using the World Health Organization criteria, as one/more of: HbA1c ≥ 48 mmol/mol, fasting plasma glucose ≥ 7.0 mmol/L, 2 h blood glucose during OGTT of ≥11.1 mmol/L, or random plasma glucose ≥ 11.1 mmol/L with symptoms of hyperglycaemia. Fasting was defined as no caloric intake for at least eight hours.

High triglyceride levels were defined as fasting serum triglycerides ≥ 1.7 mmol/L or ≥2.3 mM non-fasting, or use of triglyceride-lowering treatment.

Low HDL cholesterol levels were serum HDL ≤ 1.2 mmol/L.

### 2.5. Statistical Analysis

For women with excess adiposity (as defined above), the following were presented descriptively: baseline characteristics, pregnancy outcomes, and six-month postpartum visit results; continuous variables were presented as median and interquartile range (IQR), and categorical variables as *n* (%). Results are presented overall, and by GDM cases and non-GDM controls, and by clinical and pre-clinical obesity. Outcomes were compared between women with and without GDM, or with and without clinical obesity, using median test for continuous variables, and Chi-Square Test for categorical variables.

Among women with excess adiposity, backward multivariable logistic regression was performed to assess which factors (including GDM) contributed to clinical obesity in patients with excess adiposity. Prior to the regression analysis, continuous variables (e.g., maternal age and BMI) were centred by subtracting the median from each value. In the first step, we included maternal demographics, variables from medical history, and pregnancy outcomes. In the second step, backward stepwise logistic regression was carried out using biologically relevant variables and those with *p* < 0.1 in the backward elimination step. The relative effect of each variable was calculated as an odds ratio (OR) and its 95% confidence interval (CI). Model fit was taken to be good if the *p* value were >0.05 by the Hosmer-Lemeshow test.

Finally, as most organ dysfunction used as criteria for clinical obesity are not specific to excess adiposity, and may be secondary to other conditions (e.g., genetics, other diseases), we determined the prevalence of clinical obesity that may be causal, by calculating the prevalence of organ dysfunction in clinical obesity minus the prevalence of the same organ dysfunction among GDM and non-GDM controls with BMI < 30 kg/m^2^ (who had not otherwise been included in the previous analyses).

All analyses were performed using SPSS statistical software (version 29) and STATA ver15.0. All tests were two-sided, and *p* < 0.05 was considered statistically significant.

## 3. Results

Of 2088 women invited for postnatal review, 1086/1442 (75.3%) GDM and 562/646 (87.0%) non-GDM women attended, at a median of 5.8 (interquartile range 4.8–6.7) months after birth. A total of 391/1086 (36.0%) GDM cases had a BMI ≥ 30 kg/m^2^, compared with 120/562 (21.4%) of non-GDM controls. Excess adiposity was confirmed by WHR > 0.5 in 502 women, 385/391 (98.5%) GDM cases, and 117/120 (97.5%) non-GDM controls.

### 3.1. Demographics and Clinical Characteristics of the Cohort

Table 1 presents baseline characteristics and pregnancy outcomes, for women with excess adiposity overall, according to GDM (or not) in the most recent pregnancy, with those for women with clinical or pre-clinical obesity presented in Supplementary Table S1.

Overall, women were about 35 years of age, about 43% self-identified as being of White ethnicity, 5% of women had prior chronic hypertension, about half had a family history of diabetes, 5% a family history of pre-eclampsia, and among parous women, 28% had a history of GDM, and 9% a history of pre-eclampsia (Table 1). Six percent conceived by assisted reproductive technology, 38% were nulliparous, 16% developed either gestational hypertension or pre-eclampsia, the median gestational age at birth was 39 weeks, and 14% of neonates were large-for-gestational age. The prevalence of clinical obesity (i.e., excess adiposity with organ dysfunction) was 58.8% (295/502). Raised arterial BP was the most common criterion for clinical obesity, followed by the renal and cardiovascular (CV) clusters. No women had intracranial hypertension, reduced lung and diaphragmatic compliance causing respiratory problems, recurrent venous thromboembolism, heart failure [systolic or diastolic], pulmonary hypertension, atrial fibrillation, musculoskeletal problems, lymphoedema, or limitations of activities of daily living.

**Figure 1:**
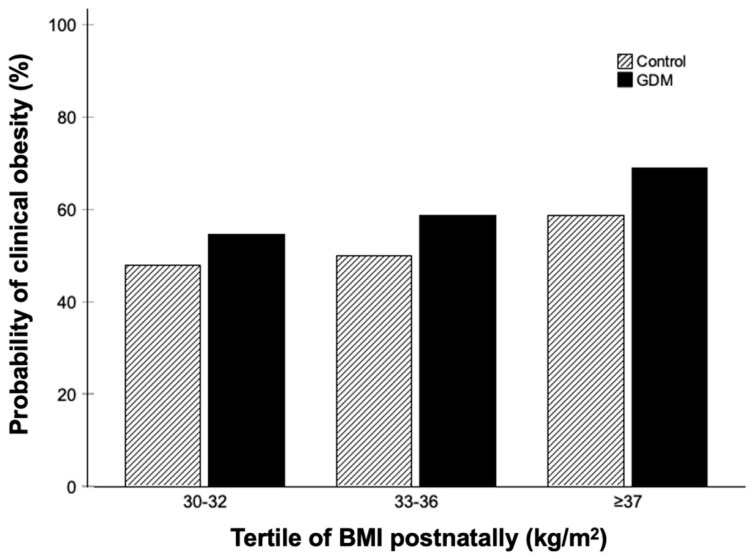
Probability of clinical obesity (%) among 502 postpartum women with excess adiposity *, according to development of GDM or not in the index pregnancy, divided by postnatal BMI (kg/m^2^) tertiles. * Obesity was defined as excess obesity: BMI ≥ 30 kg/m^2^ and a waist-to-height ratio > 0.5 and designated as pre-clinical or clinical depending on the absence or presence (respectively) of one or more cardiometabolic criteria available in this cohort.

Among GDM cases (vs. non-GDM controls), there was a higher prevalence of: Black ethnicity, family history of diabetes, multiparity and a diagnosis of GDM and/or preeclampsia in the previous pregnancy, and earlier median gestational age at birth (by 0.6 weeks) (Table 1). The prevalence of organ dysfunction among these women with excess adiposity was numerically greater, but statistically similar, among GDM cases (235/385, 61.0%) and non-GDM controls (60/117, 51.3%; *p* = 0.060), across tertiles of postnatal BMI (Figure 1). While raised arterial BP and the renal cluster were the most common criteria for clinical obesity among GDM cases and non-GDM controls, few non-GDM controls met criteria for the metabolism cluster and ART was more common than was the CV cluster.

Women with clinical (vs. pre-clinical) obesity more often had a history of chronic hypertension, conceived by ART, multiparity and a diagnosis of GDM in the previous pregnancy, developed gestational hypertension or pre-eclampsia in their most recent pregnancy, gave birth a median of 0.2 weeks earlier, and had higher median BMI at the postnatal clinic visit (Supplementary Table S1). While, by definition, women with pre-clinical obesity did not meet the Commission’s cluster criteria for clinical obesity, there were substantial minorities of women with dyslipidaemia (particularly low HDL, 29.5%), dysglycaemia (particularly pre-diabetes, 38.6%), and reduced eGFR (11.6%).

### 3.2. Predictors of Clinical (vs. Pre-Clinical) Obesity

Table 2 shows the univariable and multivariable logistic regression analysis performed to characterize clinical (vs. pre-clinical) obesity. Clinical obesity was associated with higher maternal age, multiparity with GDM in a prior pregnancy (before the index pregnancy), development of preeclampsia or gestational hypertension in the most recent (index) pregnancy, and BMI ≥ 35 kg/m^2^ at the postnatal visit. The AUC ROC was 0.644 (95% CI 0.595–0.692) (Supplementary Figure S1). Model fit was good (Hosmer-Lemeshow test *p* = 0.452).

Table 3 includes the 1137 with BMI < 30 kg/m^2^ (695 with prior GDM and 442 without). The prevalence of organ dysfunction criteria that define clinical obesity among the 1137 participants with BMI < 30 kg/m^2^ was 35.9% (408/1137), and among those with clinical obesity was 58.8% (295/502, as presented in Table 1). As for women with excess adiposity (i.e., BMI ≥ 30 kg/m^2^ with WHR > 0.50), the most common end-organ dysfunction was raised arterial BP, although those with BMI < 30 kg/m^2^ more often met the ART cluster criterion, and those with clinical obesity more often met the CV cluster criterion. The obesity-adjusted prevalence of organ dysfunction related to excess adiposity was 22.9% (i.e., 58.8% minus 35.9%).

## 4. Discussion

### 4.1. Summary of Findings and Interpretation

Our findings suggest that in a population of postpartum women following GDM and their non-GDM controls, excess adiposity may be common, just over half of these women have end-organ dysfunction, and up to 1/3 of that is potentially associated with obesity (i.e., clinical obesity) and may be modifiable with weight loss. Importantly, clinical obesity in our cohort has limited predictability from available baseline pregnancy characteristics, pregnancy outcomes, or BMI at six months postpartum, and for one in five of these women with excess adiposity, the relationship between adiposity and end-organ dysfunction may be causal, and therefore, amenable to obesity treatment. Furthermore, clinically important minorities of women with excess adiposity who do not meet criteria for clinical obesity, have metabolic and renal abnormalities of relevance to their future health.

While our findings indicate associations between obesity and organ dysfunction, it is important to note that obesity was defined using a BMI threshold of ≥30 kg/m^2^ without accounting for ethnic-specific cut-offs. Consequently, the burden of adiposity-related risk may be underestimated in populations for whom lower BMI thresholds are recommended.

### 4.2. Comparison with Literature

It is noteworthy that postpartum follow-up in our study population was over 75%, and much higher than the 19–73% reported by others [19], even in recent studies [20]. By systematic review (55 studies), many barriers to postnatal follow-up after GDM have been identified, at multiple levels: individual (e.g., self-efficacy), care-provider (e.g., lack of familiarity with guidelines), health system (e.g., compartmentalisation of care), and societal (e.g., social support). Therefore, it is unsurprising that in another systematic review (11 studies of various design, 3 systematic reviews), there appeared to be no single intervention that has been successful in achieving good follow-up rates across settings [21]; reminders to both women (as in our study) and healthcare professionals showed the greatest effectiveness. Also, as in our setting, others have found that shifting responsibility for scheduling the postpartum visit from the women to clinic personnel improves follow-up [22].

The recent Lancet Commission defined clinical and pre-clinical obesity to reflect the nuanced impact of excess adiposity at the individual-level, and in doing so, facilitate a medically meaningful approach to obesity care. Our findings support implementation of this diagnostic framework in the clinical assessment of postpartum women, distinguishing between those with ongoing disease (clinical obesity) and those with a variable level of health risk without illness (pre-clinical obesity), although it appears that this approach would require systematic assessment of the signs and symptoms of clinical obesity, which is not yet part of standard clinical practice. Identifying individuals with clinical obesity can help with the choice and prioritization of available anti-obesity interventions [3]. This approach aligns with EOSS [1] and the EASO framework [2]. While lifestyle interventions may play a greater role in decreasing overall health risk for women with pre-clinical obesity, these approaches are alone, likely insufficient to improve organ function and related clinical manifestations in women with clinical obesity. Qualitative work has highlighted that women find relevant changes challenging, and lower rates of compliance reduce their impact [23].

Effective therapies for obesity include pharmacological approaches, especially new glucagon-like peptide-1 (GLP-1) and GLP-1/glucose-dependent insulinotropic polypeptide (GIP) agonists [24], as well as bariatric/metabolic surgery [25]. While both approaches achieve substantial weight reduction and improve several obesity-linked conditions, no trials have yet specifically investigated the role of available therapies in achieving remission or improving the manifestations of clinical obesity.

Future work should include larger trials of metabolic surgery, and incretin-based and other therapies for clinical obesity, to build the evidence base and support clinical guidance. These studies should include testing the effects of therapies on maternal and fetal outcomes of possible future pregnancies [26]. Our findings regarding prediction of clinical obesity postpartum must be replicated, to inform postpartum care pathways.

### 4.3. Strengths and Limitations

Strengths of our study include the unselected nature of the cohort of women with GDM and controls who were approached for follow-up, and the high proportion who participated in postnatal clinic review. Our population is large for a postpartum follow-up study at almost six months after birth. Our participants were diverse, ethnically (i.e., 32% Black and 12% South Asian ethnicity) and socioeconomically, inclusive of those at greatest risk of cardio-renal-metabolic disorders. We collected all core outcomes for longer-term follow-up of GDM pregnancies beyond six weeks postpartum [27]. Our definition of clinical obesity took into account GDM and the specificity of organ dysfunction criteria for clinical obesity.

Limitations of our study include the lack of information on additional criteria for assessment of excess adiposity (such as direct measurement of body fat) that would have allowed us to more accurately exclude obesity in women with BMI < 30 kg/m^2^; as such, we may have under-estimated the prevalence of excess adiposity and clinical obesity postpartum. We have no information about factors that affect adiposity and metabolic health (particularly diet, physical activity, genetic factors, biomarkers and other confounders) that would have allowed us to adjust for residual confounding of the relationship between adiposity and clinical obesity criteria. We did not apply minority ethnic group-specific criteria for obesity (≥27.5 kg/m^2^), proposed to reflect higher cardiovascular risk in these groups at a given BMI [28], so we may have underestimated the performance of BMI relative to the detection of excess adiposity; however, this revised BMI cut-off is not universally agreed, with for example, different values for the risk of type 2 diabetes by specific ethnicity [29], and we adjusted for ethnicity in our prediction of clinical obesity. Also, we lacked information about all criteria for clinical obesity, however rare among women of reproductive age. Finally, as the study was conducted at a single tertiary care centre with a structured postpartum follow-up program and high attendance rates, the generalizability of the findings and, consequently, their external validity, should be considered when extrapolating the results.

## 5. Conclusions

Our study shows that in a cohort of postpartum women having experienced GDM and their non-GDM controls, excess adiposity is common. At least half of such women have cardio-renal-metabolic dysfunction that cannot be predicted, and at least 20% have end-organ dysfunction that is likely due to that excess adiposity. Our findings support routine postnatal follow-up and use of a clinical obesity diagnostic framework to identify subjects with more urgent need for obesity care and health monitoring.

## Figures and Tables

**Table 1:** Baseline characteristics, pregnancy outcomes, and criteria for clinical obesity, among 502 postpartum women with excess adiposity *, according to development of GDM or not in the index pregnancy. Data presented as median (interquartile range) or *n* (%), and greyed cells represent *p*-value < 0.05.

	All (*n* = 502)	Non-GDM (*n* = 117; 23.3%)	GDM (*n* = 385; 76.7%)	*p*-Value
**Demographic and pregnancy characteristics**				
Age (years)	35.0 (31.5–38.2)	34.6 (31.4–37.9)	35.0 (31.5–38.2)	0.784
Ethnicity				<0.0011
White	218 (43.4)	69 (59.0)	149 (38.7)	
Black	192 (38.2)	34 (29.1)	158 (41.0)	
South Asian	59 (11.8)	6 (5.1)	53 (13.8)	
East Asian	12 (2.4)	0 (0)	12 (3.1)	
More than one	21 (4.2)	8 (6.8)	13 (3.4)	
Chronic hypertension	23 (4.6)	4 (3.4)	19 (4.9)	0.492
1st or 2nd degree family history of diabetes mellitus	240 (47.8)	28 (23.9)	212 (55.1)	<0.001
Family history of preeclampsia	22 (4.4)	5 (4.3)	17 (4.4)	0.948
Method of conception				0.699
Spontaneous	469 (93.4)	109 (93.2)	360 (93.5)	
Ovulation induction	2 (0.4)	0 (0)	2 (0.5)	
In vitro fertilization	31 (6.2)	8 (6.8)	23 (6.0)	
Parity				0.003
Nulliparous	198 (39.4)	60 (51.3)	138 (35.8)	
Parous	304 (60.6)	57 (48.7)	247 (64.2)	
Parous with previous GDM	66 (21.7)	1 (1.8)	65 (26.3)	<0.001
Parous without previous GDM	238 (78.3)	56 (98.2)	182 (73.7)	
Parous with previous PE	20 (6.6)	2 (3.5)	18 (7.3)	0.007
Parous without previous PE	284 (93.4)	55 (96.5)	229 (92.7)	
**Pregnancy outcomes**				
GDM	385 (76.7%)	0 (0%)	385 (100%)	<0.001
PE or gestational hypertension	69 (13.8)	12 (10.3)	57 (11.4)	0.697
Gestational age at delivery (wks)	39.0 (38.3–39.6)	39.4 (38.9–40.4)	38.9 (38.0–39.4)	<0.001
Birthweight (percentile)	57.3 (25.4–79.4)	52.1 (25.3–76.0)	58.6 (25.4–80.0)	0.350
>90th percentile	67 (13.4)	14 (12.3)	53 (13.8)	0.683
**Postnatal visit**				
Interval from delivery (months)	5.6 (4.7–6.6)	6.1 (5.2–6.7)	5.4 (4.6–6.5)	<0.001
BMI at postnatal visit (kg/m^2^)	34.4 (31.9–38.2)	33.3 (31.6–36.6)	34.5 (32.0–38.6)	0.020
WHR	0.63 (0.59–0.67)	0.59 (0.55–0.64)	0.64 (0.60–0.67)	<0.001
Clinical obesity criteria †	295 (58.8)	60 (51.3)	235 (61.0)	0.060
Raised arterial BP cluster	212/295 (71.9)	47/60 (78.3)	165/235 (70.2)	0.212
Stage 1 hypertension	154/295 (52.2)	38/60 (63.3)	116/235 (49.4)	0.150
Stage 2 hypertension	54/295 (18.3)	9/60 (15.0)	45/235 (19.1)	
Antihypertensive medication	20/295 (6.8)	4/60 (6.7)	16/235 (6.8)	0.969
ART cluster	33/295 (11.2)	8/60 (13.3)	25/235 (10.6)	0.554
Metabolism cluster †	40/295 (13.6)	2/60 (3.3)	38/235 (16.2)	0.010
Dysglycaemia cluster	163/295 (55.3)	12/60 (20.0)	151/235 (64.3)	<0.001
Prediabetes	145/295 (49.2)	12/60 (20.0)	133/235 (56.6)	<0.001
Diabetes type 2	18/295 (6.1)	0/60 (0)	18/235 (7.7)	
Dyslipidaemia cluster	51/295 (17.3)	4/60 (6.7)	47/235 (20.0)	0.015
Triglycerides ≥ 1.7 mM fasting, or ≥2.3 mM non-fasting	67/295 (22.7)	7/60 (11.7)	60/235 (25.5)	0.022
HDL cholesterol ≤ 1.2 mM	110/295 (37.3)	14/60 (23.3)	96/235 (40.9)	0.012
Renal cluster	71/295 (24.1)	10/60 (16.7)	61/235 (24.9)	0.133
Microalbuminuria	71/295 (24.1)	10/60 (16.7)	61/235 (26.0)	0.133
Reduced eGFR	38/295 (12.9)	6/60 (10.0)	32/235 (13.6)	0.455
CV cluster—diastolic dysfunction §	66/295 (22.4)	3/60 (5.0)	63/235 (26.8)	<0.001

ART = assisted reproductive technology, BMI = body mass index, BP = blood pressure, CV = cardiovascular, eGFR = estimated glomerular filtration rate, GDM = gestational diabetes mellitus, HDL = high-density lipoprotein, PE = pre-eclampsia, wks = weeks, WHR = waist-to-height ratio. * Excess adiposity was defined as defined by BMI ≥ 30 kg/m^2^ and waist/height ratio > 0.5. † Clinical obesity was defined as excess adiposity and one/more available criteria defined by The Lancet Diabetes and Endocrinology Commission^1^ (see Section 2). §: Diastolic dysfunction was defined as a left ventricular filling pressures ≥90th percentile (9.6).

**Table 2:** Logistic regression model to predict clinical obesity.

	Univariable		Multivariable	
	OR (95%CI)	*p*-Value	OR (95%CI)	*p*-Value
**Baseline characteristics**				
Age—Δ1 (years)	1.05 (1.01–1.10)	0.006	1.05(1.01–1.09)	0.008
BMI at 12 weeks’ gestation—34 (kg/m^2^)	1.55 (1.08–2.22)	0.016	NA	
Ethnicity		0.238		
White	1.00			
Black	1.58 (1.06–2.36)	0.024	NA	
South Asian	1.15 (0.65–2.06)	0.632	NA	
East Asian	0.85 (0.27–2.71)	0.780	NA	
More than one	1.13 (0.46–2.79)	0.791	NA	
Chronic hypertension	Undefined		NA	
Method of conception				
Spontaneous	1.00			
Ovulation induction	Undefined		NA	
In vitro fertilization	Undefined		NA	
1st or 2nd degree family history of diabetes	1.18 (0.83–1.68)	0.368	NA	
Family history of preeclampsia	2.47 (0.90–6.81)	0.080	2.77 (0.98–7.86)	0.055
Parity				
Nulliparous	1.00			
Parous	1.24 (0.87–1.79)	0.239	NA	
Parous with previous GDM	2.13 (1.16–3.93)	0.015	4.03 (1.17–14.00)	0.028
Parous with no previous GDM	1.09 (0.74–1.59)	0.674	NA	
Parous with previous PE	3.20 (1.03–9.92)	0.044	NA	
Parous without previous PE	1.18 (0.81–1.70)	0.388	NA	
**Pregnancy outcome** (index pregnancy)				
Gestational diabetes	1.49 (0.98–2.26)	0.061	NA	
Preeclampsia or gestational hypertension	1.77 (1.02–3.09)	0.044	1.77 (1.00–3.14)	0.050
Gestational age at delivery—39 (weeks)	0.55 (0.39–0.80)	0.001	NA	
Birthweight (percentile) >90 th percentile	1.14 (0.67–1.93)	0.633	NA	
**Postnatal visit** (following index pregnancy)				
BMI at postnatal visit ≥ 35 (kg/m^2^)	1.79 (1.24–2.57)	0.002	1.71 (1.17–2.48)	0.005

BMI = body mass index, CI = confidence interval, NA = Not applicable.

**Table 3:** Prevalence of the available criteria for clinical obesity, according to BMI.

	No Obesity BMI < 30 kg/m^2^ (*n* = 1137)	Excess Adiposity * BMI ≥ 30 kg/m^2^ + WHR > 0.5 (*n* = 502)	*p*-Value
BMI at postnatal visit (kg/m^2^)	24.4 (22.0–27.1)	34.4 (31.9–38.2)	<0.001
**One or more of the following:**	408 (35.9)	295 (58.8)	<0.001
Assisted reproductive technology cluster	115 (10.1)	33 (6.6)	0.021
Metabolism cluster	11 (1.0)	40 (8.0)	<0.001
Dysglycaemia cluster	393 (34.6)	247 (49.2)	<0.001
Prediabetes	364 (32.0)	225 (44.8)	<0.001
Diabetes type 2	29 (2.6)	22 (4.4)	<0.001
Dyslipidaemia cluster	28 (2.5)	61 (12.2)	<0.001
Triglycerides ≥ 1.7 mM if fasting and ≥2.3 mM if not fasting	60 (5.3)	87 (17.3)	<0.001
HDL cholesterol ≤ 1.2 mM	170 (15.0)	171 (34.1)	<0.001
Renal cluster	120 (10.6)	71 (14.1)	0.037
Microalbuminuria	118 (10.4)	71 (14.1)	0.028
Reduced eGFR	84 (7.4)	62 (12.4)	0.001
Raised arterial BP cluster	194 (17.1)	212 (42.2)	<0.001
Stage 1 hypertension	163 (14.3)	154 (30.7)	<0.001
Stage 2 hypertension	26 (2.3)	54 (10.8)	<0.001
Antihypertensive medication	10 (0.9)	20 (4.0)	<0.001
Cardiovascular cluster—diastolic dysfunction (Mitral valve E/e’)	51 (4.5)	66 (13.1)	<0.001

BMI = body mass index, BP = blood pressure, eGFR = estimated glomerular filtration rate, HDL = high-density lipoprotein, PE = pre-eclampsia, wks = weeks, WHR = waist-to-height ratio. * The 9 participants without obesity, based on BMI ≥ 30 kg/m^2^ and WHR ≤ 0.5, are not presented, as the small number would make comparison with other categories uninformative.

## Data Availability

The original contributions presented in the study are included in the article/Supplementary Material, further inquiries can be directed to the corresponding author.

## Supplementary Materials

The following supporting information can be downloaded at: https://www.mdpi.com/article/10.3390/nu18020212/s1, Table S1. Baseline characteristics, pregnancy outcomes, and criteria for clinical obesity, among 502 postpartum women with excess adiposity, according to pre-clinical or clinical obesity. Figure S1. Receiver operating curve for the prediction of clinical obesity at the 5-month postnatal visit.

## Abbreviations

The following abbreviations are used in this manuscript:

GDM	Gestational diabetes
BMI	Body mass index
AUC	Area under the curve
CI	Confidence interval
EOSS	Edmonton Obesity Staging System
EASO	European Association for the Study of Obesity
NICE	National Institute for Health and Care Excellence
OGTT	Oral glucose tolerance test
BP	Blood pressure
WHR	Waist-to-height ratio
uACR	Urine albumin/creatinine ratio
E/e’	Ratio of early mitral inflow velocity (E) to early diastolic mitral annular velocity (e’)
eGFR	Estimate glomerular filtration rate
DM	Diabetes mellitus
IQR	Interquartile range
OR	Odds ratio
CV	Cardiovascular
PE	Pre-eclampsia
ART	Assisted reproductive technology
HDL	High-density lipoprotein
GLP-1	Glucagon-like peptide-1
GLP-1/GIP	Glucagon-like peptide-1/glucose-dependent insulinotropic polypeptide
