# Supplementary material for: Prevalence of Clinical and Pre-Clinical Obesity at Six Months Postpartum Following Gestational Diabetes Mellitus
Source: Nutrients. 2026 Jan 9;18(2):212. doi: 10.3390/nu18020212 (PMC12845492; doi:10.3390/nu18020212)
Supplement: Supplementary file 1 [file nutrients-18-00212-s001.zip › nutrients-4051197-supplementary.pdf]

Supplementary Table S1. Baseline characteristics, pregnancy outcomes, and criteria for clinical obesity, among 502 postpartum women with excess adiposity*, according to pre-clinical or clinical obesity. Data presented as median (interquartile range) or n (%), and greyed cells represent p-value <0.05.

	All (n=502)	Pre-clinical obesity (n=207; 41.2%)	Clinical obesity (n=295; 58.8%)	p-value
**Demographic and pregnancy characteristics**				
Age (years)	35.0 (31.5-38.2)	34.8 (30.9-37.8)	35.1 (32.0-38.8)	0.495
Ethnicity				0.235
White	218 (43.4)	100 (48.3)	118 (40.0)	
Black	192 (38.2)	67 (32.4)	125 (42.4)	
South Asian	59 (11.8)	25 (12.1)	34 (11.5)	
East Asian	12 (2.4)	6 (2.9)	6 (2.0)	
More than one	21 (4.2)	9 (4.3)	21 (4.1)	
Chronic hypertension	23 (4.6)	0 (0)	23 (7.8)	<0.001
1st or 2nd degree family history of diabetes mellitus	240 (47.8)	94 (45.4)	146 (49.5)	0.368
Family history of preeclampsia	22 (4.4)	5 (2.4)	17 (5.8)	0.071
Method of conception				<0.001
Spontaneous	469 (93.4)	207 (100.0)	262 (88.8)	
Ovulation induction	2 (0.4)	0 (0)	2 (0.7)	
In vitro fertilization	31 (6.2)	0 (0)	31 (10.5)	
Parity				0.238
Nulliparous	198 (39.4)	88 (42.5)	110 (37.3)	
Parous	304 (60.6)	119 (57.5)	185 (62.7)	
Parous with previous GDM	66 (21.7)	18 (15.1)	48 (25.9)	0.043
Parous without previous GDM	238 (78.3)	101 (84.9)	137 (74.1)	
Parous with previous PE	20 (6.6)	4 (3.4)	16 (8.7)	0.099
Parous without previous PE	284 (93.4)	115 (96.6)	169 (91.3)	
**Pregnancy outcomes**				
GDM	385 (76.7%)	150 (72.5)	235 (79.7)	0.060
PE or gestational hypertension	69 (13.8)	20 (9.7)	47 (15.9)	0.020
Gestational age at delivery (wks)	39.0 (38.3-39.6)	39.1 (38.6-39.9)	38.9 (38.0-39.4)	0.020
Birthweight (percentile)	57.3 (25.4-79.4)	60.5 (29.5-79.9)	53.6 (22.6-79.9)	0.190
>90th percentile	67 (13.4)	26 (12.6)	41 (14.0)	0.633
**Postnatal visit**				
Interval from delivery (months)	5.6 (4.7-6.6)	5.6 (4.7-6.7)	5.7 (4.7-6.5)	0.999
BMI at postnatal visit (kg/m^2)	34.4 (31.9-38.2)	33.5 (31.6-37.4)	34.9 (32.1-39.0)	0.011
WHR	0.63 (0.59-0.67)	0.62 (0.58-0.66)	0.64 (0.59-0.68)	<0.001
Clinical obesity criteria †	295 (58.8)	0 (0%)	295 (100%)	<0.001
Raised arterial BP cluster	212/295 (71.9)	0/207 (0)	212/295 (71.9)	<0.001
Stage 1 hypertension	154/295 (52.2)	0/207 (0)	154/295 (52.2)	<0.001
Stage 2 hypertension	54/295 (18.3)	0/207 (0)	54/295 (18.3)	
Antihypertensive medication	20/295 (6.8)	0/207 (0)	20/295 (6.8)	0.002
ART cluster	33/295 (11.2)	0 (0)	33/295 (11.2)	<0.001
Metabolism cluster†	40/295 (13.6)	0/207 (0)	40/295 (13.6)	<0.001
Dysglycaemia cluster	163/295 (55.3)	84/207 (40.6)	163/295 (55.3)	0.001
Prediabetes	145/295 (49.2)	80/207 (38.6)	145/295 (49.2)	0.002
Diabetes type 2	18/295 (6.1)	4/207 (1.9)	18/295 (6.1)	

Dyslipidaemia cluster	51/295 (17.3)	10/207 (4.8)	51/295 (17.3)	<0.001
Triglycerides ≥1.7mM fasting, or ≥2.3mM non-fasting	67/295 (22.7)	20/207 (9.7)	67/295 (22.7)	<0.001
HDL cholesterol ≤1.2mM	110/295 (37.3)	61/207 (29.5)	110/295 (37.3)	0.069
Renal cluster	71/295 (24.1)	0/207 (0)	71/295 (24.1)	<0.001
Microalbuminuria	71/295 (24.1)	0/207 (0)	71/295 (24.1)	<0.001
Reduced eGFR	38/295 (12.9)	24/207 (11.6)	38/295 (12.9)	0.666
CV cluster – diastolic dysfunction§	66/295 (22.4)	0/207 (0)	66/295 (22.4)	<0.001

*ART = assisted reproductive technology, BMI = body mass index, BP = blood pressure, CV = cardiovascular, eGFR = estimated glomerular filtration rate, GDM = gestational diabetes mellitus, HDL = high-density lipoprotein, PE = pre-eclampsia, wks = weeks, WHR = waist-to-height ratio. * Excess adiposity was defined as defined by BMI ≥ 30 kg/m2 and waist/height ratio > 0.5. † Clinical obesity was defined as excess adiposity and one/more available criteria defined by The Lancet Diabetes and Endocrinology Commission1 (see Section 2). §: Diastolic dysfunction was defined as a left ventricular filling pressures ≥90th percentile (9.6).*

Supplementary Figure S1. Receiver operating curve for the prediction of clinical obesity at the 5-month postnatal visit*.

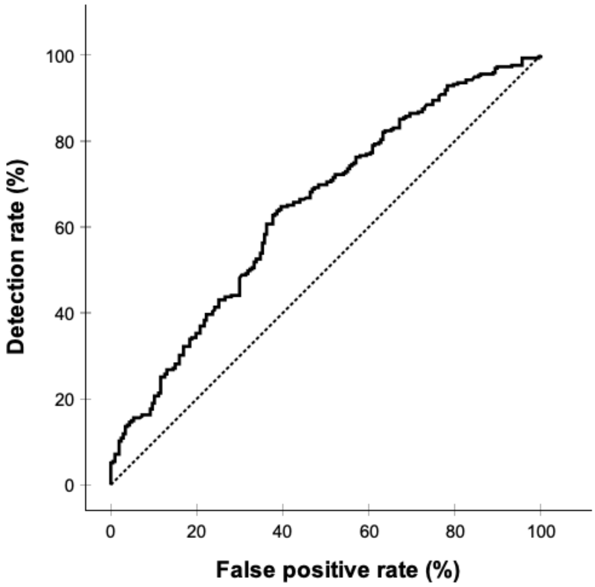

* The calibration of the model was done by Hosmer-Lemeshow test (p=0.452) and the discrimination was AUC=0.644 (0.595-0.692)
